# Supplementary material for: Causal Relationship between Aging and Anorexia Nervosa: A White-Matter-Microstructure-Mediated Mendelian Randomization Analysis
Source: Biomedicines. 2024 Aug 16;12(8):1874. doi: 10.3390/biomedicines12081874 (PMC11351342; doi:10.3390/biomedicines12081874)
Supplement: Supplementary file 1 [file biomedicines-12-01874-s001.zip › Supplementary Figure/Figure S8/Leaveoneout plot of MR analysis for TL to ubm-b-1773.pdf]

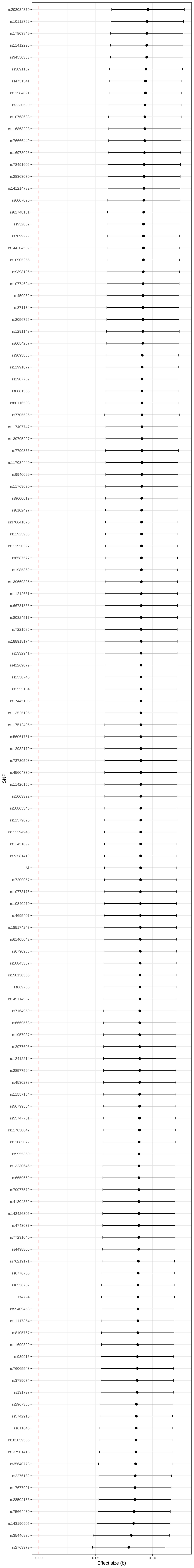
rs202034370
rs10112752
rs17803849
rs11412296
rs34550383
rs3891167
rs4731541
rs11584821
rs2230590
rs10768683
rs116863223
rs76666449
rs16978028
rs78491606
rs28363070
rs141214782
rs6007020
rs61748181
rs932002
rs7099229
rs144204502
rs10905255
rs9398196
rs10774624
rs450962
rs871134
rs2056726
rs1291143
rs6054257
rs3093888
rs11991877
rs1907702
rs6881568
rs80116508
rs7705526
rs117407747
rs139795227
rs7790856
rs117034449
rs9940099
rs11769630
rs9600019
rs8102497
rs376641875
rs12925933
rs111950327
rs6587577
rs1985369
rs139669835
rs11212631
rs66731853
rs80324517
rs7221585
rs188918174
rs1332941
rs41269079
rs2538745
rs2555104
rs17445108
rs113525195
rs117512405
rs56061761
rs12932179
rs73730598
rs45604339
rs11426156
rs1003322
rs10805346
rs11579626
rs112394943
rs12451892
rs73581419
All
rs7209057
rs10773176
rs10840270
rs4695407
rs185174247
rs61405042
rs6790988
rs10845387
rs150150565
rs869785
rs145114957
rs7164950
rs6669563
rs1957937
rs2977608
rs12412214
rs28577594
rs4530278
rs11557154
rs56799554
rs55747751
rs117630647
rs11085072
rs9955360
rs13230646
rs6659669
rs79977579
rs41304832
rs142426306
rs4743037
rs77231040
rs4498805
rs76219171
rs6776756
rs6536702
rs4724
rs59409453
rs11117354
rs8105767
rs11699829
rs939916
rs76065543
rs3785074
rs131797
rs2967355
rs5742915
rs611646
rs182059586
rs137901416
rs35640778
rs2276182
rs17677991
rs28502153
rs75664430
rs143190905
rs35446936
rs2763979
SNP
0.00
0.05
0.10
Effect size (b)
